# Impedimetric Sensor for SARS-CoV-2 Spike Protein Detection: Performance Assessment with an ACE2 Peptide-Mimic/Graphite Interface

**DOI:** 10.3390/bios14120592

**Published:** 2024-12-03

**Authors:** Diego Quezada, Beatriz Herrera, Rodrigo Santibáñez, Juan Luis Palma, Esteban Landaeta, Claudio A. Álvarez, Santiago Valenzuela, Kevin Cobos-Montes, David Ramírez, Paula A. Santana, Manuel Ahumada

**Affiliations:** 1 Instituto de Ciencias Aplicadas, Facultad de Ingeniería, Universidad Autónoma de Chile, el Llano Subercaseaux 2801, San Miguel, Santiago 8910060, Chile; diego.quezada@uautonoma.cl (D.Q.); beatriz.herrera@cloud.uautonoma.cl (B.H.); rodrigo.santibanez@cloud.uautonoma.cl (R.S.); s.valenzuelamejias@icloud.com (S.V.); 2 School of Engineering, Universidad Central de Chile, Santiago 8330601, Chile; juan.palma@ucentral.cl (J.L.P.); esteban.landaeta@ucentral.cl (E.L.); 3 Center for the Development of Nanoscience and Nanotechnology (CEDENNA), Santiago 9170124, Chile; 4 Laboratorio de Cultivo de Peces, Departamento de Acuicultura, Universidad Católica del Norte, Coquimbo 1781421, Chile; claudio.alvarez@ucn.cl; 5 Laboratorio de Fisiología y Genética Marina, Centro de Estudios Avanzados en Zonas Áridas, Larrondo 1281, Coquimbo 1781421, Chile; 6 Departamento de Ciencias Químicas, Facultad de Ciencias Exactas, Universidad Andrés Bello, Sede Concepción, Talcahuano 4260000, Chile; k.cobosmontes@uandresbello.edu; 7 Departamento de Farmacología, Facultad de Ciencias Biológicas, Universidad de Concepción, Concepción 4030000, Chile; dramirezs@udec.cl; 8 Escuela de Biotecnología, Facultad de Ciencias, Ingeniería y Tecnología, Universidad Mayor, Camino La Pirámide 5750, Huechuraba, Santiago 8580745, Chile; 9 Centro de Nanotecnología Aplicada, Facultad de Ciencias, Ingeniería y Tecnología, Universidad Mayor, Camino La Pirámide 5750, Huechuraba, Santiago 8580745, Chile

**Keywords:** ACE2 peptide-mimic, biosensors, impedimetric detection, graphite surface, spike protein detection

## Abstract

The COVID-19 pandemic has prompted the need for the development of new biosensors for SARS-CoV-2 detection. Particularly, systems with qualities such as sensitivity, fast detection, appropriate to large-scale analysis, and applicable in situ, avoiding using specific materials or personnel to undergo the test, are highly desirable. In this regard, developing an electrochemical biosensor based on peptides derived from the angiotensin-converting enzyme receptor 2 (ACE2) is a possible answer. To this end, an impedimetric detector was developed based on a graphite electrode surface modified with an ACE2 peptide-mimic. This sensor enables accurate quantification of recombinant 2019-nCoV spike RBD protein (used as a model analyte) within a linear detection range of 0.167–0.994 ng mL^−1^, providing a reliable method for detecting SARS-CoV-2. The observed sensitivity was further demonstrated by molecular dynamics that established the high affinity and specificity of the peptide to the protein. Unlike other impedimetric sensors, the herein presented system can detect impedance in a single frequency, allowing a measure as fast as 3 min to complete the analysis and achieving a detection limit of 45.08 pg mL^−1^. Thus, the proposed peptide-based electrochemical biosensor offers fast results with adequate sensitivity, opening a path to new developments concerning other viruses of interest.

## 1. Introduction

Infectious diseases can originate in one region and spread rapidly to other parts of the planet due to constant human mobility, potentially leading to a pandemic such as COVID-19. A key to preventing and controlling the infection spread is the development of diagnostic methods that can quickly identify the pathogen and, thus, adequately treat the infection. However, the most common laboratory methods for detecting microorganisms are based on two main approaches: detection of genetic material using the polymerase chain reaction (PCR) or detection of antigens, the main proteins, through immunosorbent assay linked to enzymes (ELISA) [1]. Although both strategies offer high sensitivity and specificity, they require highly trained personnel, sophisticated equipment, and complex sample handling, which increases the analysis costs. Further, the requirements to carry out these methods make it difficult to analyze samples in situ through these methodologies. For that reason, the new diagnostic technologies are focused on developing biosensors, whose integrated devices allow the biomolecular recognition of analytes in situ, which depends on the interaction of the immobilized biomolecules and the target analyte. Then, these biological reactions are converted into measurable signals [2,3].

Before the COVID-19 pandemic, biosensors for viral pathogens were developed against influenza viruses. Various studies describe the use of DNA probes to detect viral genetic material [4,5,6,7], the use of lectins for the detection of surface protein glycosylations [8,9], as well as the development of antibodies that are immobilized in the devices to recognize the hemagglutinin and neuraminidase proteins of different influenza strains [10,11,12], whose interactions are then mostly converted into electrochemical signals. Regarding this last strategy, the different types of non-covalent bonds between amino acids, such as hydrogen bonding, van der Waals forces, π-stacking, and hydrophobic and electrostatic interactions, make it convenient to develop these technologies based on the biorecognition of proteins from pathogen surfaces. In addition, the use of synthetic peptides provides great advantages in replacing antibodies for the development of electrochemical biosensors [13]. Since these can be designed and synthesized, it is possible to modify specific functional groups, increasing their stability and selectivity towards a target analyte. Another point in favor of using peptides is that they can be used as anti-biofouling agents, a recurring problem in electrochemical biosensors caused by the accumulation of biomolecules on the surfaces of these devices [13].

A relevant aspect in the assembly of electrochemical devices using peptides is considering the physical and chemical properties of the amino acids that compose them. This increases their covalent or non-covalent immobilization (physical adsorption) to the surface transducer [14]. The most frequent transducers that employ peptides are gold and silica, mainly using covalent bonds of the thiol group of cysteines [14,15,16]. Yet, new approaches to developing graphene–peptide biosensors have allowed the establishment of different strategies that will enable peptides to be conjugated to the functional groups on the surface of this material. One of the most used procedures for conjugating peptides to graphene is pyrene-based conjugation, which first requires modification of the graphene surface by pyrene-based N-hydroxysuccinimide ester (NHS) to bind the peptide covalently by an amide bond [17,18,19]. In comparison, the (1-Ethyl-3-(3-dimethyl aminopropyl)carbodiimide)(EDC)/NHS strategy functionalizes the peptides to the graphene surface through the peptide amine groups [17,20]. Both peptide–graphene covalent binding strategies can provide high stability for biosensor devices.

In recent years, peptide-based biosensors have emerged as a promising approach for the detection of a wide range of analytes [21], including DNA/RNA [21,22,23], virus [24,25] cells [26], and proteins [21], such as enzymes involving proteases and kinases [13], antibodies [27,28], and other proteins [29]. One area where peptide-based biosensors have shown particular potential is detecting viral pathogens, including the SARS-CoV-2 virus responsible for the COVID-19 pandemic [30,31].

Given the urgent need for rapid and accurate testing methods for detecting viral diseases such as SARS-CoV-2, peptide-based biosensors are attractive for their high sensitivity, specificity, and fast response times [30,32,33]. These biosensors leverage peptides as recognition elements, which can selectively bind to target proteins, such as the spike protein [30,32,33,34], allowing for the detection of specific viral proteins associated with SARS-CoV-2 infection.

**Figure 1:**
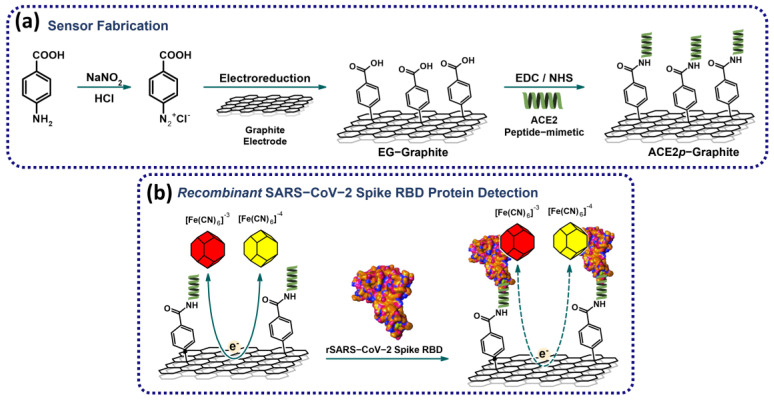
Simplified workflow of the (**a**) fabrication of the biosensor based on ACE2 peptide-mimetic and (**b**) recombinant SARS-CoV-2 spike RBD protein detection. The modification of the graphite surface is achieved by incorporating -COOH residues, which serve as anchors for the immobilization of the ACE2 peptide-mimetic (ACE2*p*). The recombinant SARS-CoV-2 RBD protein is detected by measuring the system’s total impedance using [Fe(CN)_6_]^−3/−4^ as a redox probe.

In this context, this study aims to develop an angiotensin-converting enzyme receptor 2 (ACE2) peptide mimic-based electrochemical biosensor for detecting SARS-CoV-2 spike protein (RBD), an important target for developing diagnostic and therapeutic strategies. Figure 1 summarizes the experimental workflow. The biosensor utilizes a modified electrode and a total impedance measurement strategy for rapid and sensitive detection of the target protein. Further, molecular dynamics tools are employed to understand the peptide–protein interaction and biosensor sensibility. Using peptide-based biosensors for detecting SARS-CoV-2 spike protein represents a promising approach for developing cost-effective and efficient diagnostic tools for the ongoing COVID-19 pandemic.

## 2. Materials and Methods

### 2.1. Peptide-Mimic Design and Modeling

The ACE2 peptide-mimic (ACE2*p*) was modeled considering the previously established peptide sequence [35,36] and using the Build Peptide module in Maestro–Schrödinger, using the ACE2 (PDB-ID: 6CS2—chain D) [37] as a template. The percentage identity of the peptide-mimic compared to ACE2 was evaluated using the Jalview program with the ClustalO alignment method [38]. For docking simulations, the Receptor Binding Domain (RBD) of the spike protein (PDB-ID: 6CS2—chain B) was prepared, covering the fragment from R315 to T517, representing the contact region of the RBD-ACE2 complex. The protein was prepared and minimized using the Protein Preparation Wizard, removing water molecules, ions, metals, and ligands. The missing fragment (S315-T517) was modeled using the spike sequence (UniProt-ID: P59594). Missing hydrogens were added, and ionizable residues were protonated at pH 7.4 using PropKa version 3.1.

### 2.2. Molecular Dynamics Simulations and Clustering

Following the peptide-mimic modeling, molecular dynamics simulations were performed using Desmond software with the Optimized Potentials for Liquid Simulations 4 (OPLS4) force field [39], designed to describe atomic interactions in biological and molecular systems accurately. The peptide-mimic was solvated in a cubic box of 238.328 Å^3^ using the Simple Point Charge (SPC) water model. The system was neutralized by adding Na^+^ or Cl^−^ ions, and the final NaCl concentration was adjusted to 0.15 M. The system was then relaxed using Desmond’s default relaxation protocol and equilibrated with a restraining force of 5.0 kcal × mol^−1^ × Å^−2^, applied to the peptide-mimic backbone. Equilibration was set for 20 ns under constant pressure (1 atm), number of particles, and temperature (300 K) conditions (NPT ensemble) using the Nose–Hoover isothermal–isobaric ensemble [40]. The production phase was performed without restraints for 500 ns under the same conditions. Upon completion of the simulation, a clustering analysis based on Root Mean Square Deviation (RMSD) was performed using a 2.5 Å threshold with the trj_cluster.py script from Maestro–Schrödinger. Finally, one conformer was extracted from the four most populated clusters and used in the molecular docking simulations.

### 2.3. Docking Simulations

Molecular docking simulations were conducted using the HADDOCK2.2 server [41], employing the RBD protein as the receptor and each selected ACE2 peptide-mimic conformer as the ligand. The active residues of the RBD protein were defined as those in contact with ACE2 (within a 5 Å radius) based on the crystallographic structure of the spike–ACE2 complex (PDB-ID: 6CS2). The top 200 docking solutions were clustered based on the fraction of common contacts (FCC) and ranked according to the HADDOCK score. The best docking solution from each cluster was selected for interaction analysis using the Protein–Ligand Interaction Profiler (PLIP) software [42].

### 2.4. Reagents and Solutions

Sodium nitrite (NaNO_2_), 4-aminobenzoic acid (4-ABA), N-hydroxysuccinimide (NHS), N-(3-dimethylaminopropyl)-N′-ethylcarbodiimide (EDC), hydrochloric acid (HCl), potassium ferrocyanide (K_4_[Fe(CN)_6_]), potassium ferricyanide (K_3_[Fe(CN)_6_]), bovine serum albumin (BSA) and recombinant 2019-nCoV spike RBD protein (SP) (code SAB5700590) were purchased from Sigma-Aldrich, St. Louis, MO, USA and used without any further purification. Phosphate-buffered saline (PBS) solution 10× Molecular Biology Grade was purchased from Corning Life Sciences (Corning, NY, USA).

Aqueous solutions were prepared using ultrapure water (18.2 MΩ cm) from Milli-Q Systems (Millipore Inc., Burlington, MA, USA). PBS 1× pH 5.0 and pH 7.4 solutions were obtained by adjusting the acidity of a diluted PBS solution (1×).

### 2.5. Apparatus and Electrodes

AFM characterization was carried out using a JUPITER XR microscope in tapping mode, with an AC240 tip from Asylum Research utilizing a curvature radius of 7 nm.

Scanning electron microscopy (SEM) coupled to energy dispersive X-ray spectroscopy (EDS) was carried out in a Phenom ProX (ThermoFisher, Waltham, MA, USA) at 15 kV.

The electrochemical experiments reported herein were performed in a PalmSens EmStat4S potentiostat/galvanostat. All measurements were carried out using a three-electrode system with an Ag/AgCl 3M reference electrode and a platinum coil as a counter electrode, purchased from CH Instruments, Bee Cave, TX, USA.

Graphite disks used as working electrodes were fabricated from a 1 mm thick graphite plate purchased from Wuhu Yanjiao Electronic Commerce Co., Anhui, China; they were cut into disks with a diameter of 1.5 cm. Working electrodes were polished until mirror finishing prior to any measurement or any other modification procedure.

### 2.6. ACE2 Peptide-Mimic Synthesis

In a previous study, Zhang et al. [35] established and synthesized an ACE2 peptide-mimic (ACE2*p*) that interacted with the spike protein. Based on that report, a slightly modified ACE2*p* was designed to selectively bind to the SARS-CoV-2 spike protein [36]. To this end, the ACE2*p* was assembled manually in plastic syringes fitted with a porous polyethylene disc, using the Fmoc/t-butyl strategy following a previously reported synthesis method [36]. Briefly, the peptide was prepared on Rink amide resin (Iris) (0.65 mmol g^−1^ substitution). Then, it was cleaved from the resin by treating it with trifluoroacetic acid solution (TFA/H_2_O/triisopropylsilane/ethanedithiol) (92.5:2.5:2.5:2.5) (*v*/*v*/*v*/*v*) for 90 min at room temperature, precipitated with diethyl ether, extracted with Milli-Q water, and lyophilized.

### 2.7. Preparation of Electrografted Graphite Electrodes

The electrografted graphite (EG–graphite) electrode was prepared following a previously reported method [36]. Briefly, a 2 mM solution of NaNO_2_ and 2 mM of 4-ABA in HCl 0.5 M was prepared and bubbled with N_2_ gas for 10 min. The electrode modification was achieved by performing four cyclic voltammetry scans between 0.2 V and −0.7 V at 50 mVs^−1^; the electrodes were then rinsed with Milli-Q water and dried under N_2_ current.

### 2.8. Preparation of Peptide-Modified Graphite Electrode

Immobilization of the ACE2*p* was performed in three steps. First, activation of the terminal carboxylic groups of the EG–graphite surface was carried out by incubating the electrode in PBS 1× (pH 5.0) containing 100 mM EDC and 20 mM NHS for 1 h at room temperature; after that, the electrodes were washed with PBS 1× (pH 5.0). The second step involved incorporating the ACE2*p*; for this, the electrodes were incubated with 10 μg mL^−1^ ACE2 *p* solution in PBS 1× pH 7.4. for 3 h. The electrodes were then washed with PBS pH 7.4. Finally, the free sites on the electrode surface were blocked by incubating the electrode in a 0.1% BSA in PBS 1× pH 7.4 for 1 h. The ACE2*p*-modified graphite electrodes (ACE2*p*–graphite) were then washed with PBS 1× pH 7.4 and stored under an N_2_ atmosphere.

Cyclic voltammetry, AFM, and SEM/EDX analyses were performed to confirm the modification of the electrodes.

### 2.9. Detection Procedure

Recombinant 2019-nCoV spike RBD protein (SP) was detected using electrochemical impedance spectroscopy (EIS) at open circuit potential with an amplitude of 5 mV between 0.1 Hz and 10 kHz. Measurements were carried out in a 200 μL electrochemical cell filled with a 5.0 mM ferri/ferrocyanide [Fe(CN)_6_]^4−/3−^ solution in PBS 1× pH 7.4 as blank, and detection of SP was performed by adding fixed amounts of SP. Calibration curves were constructed using the total impedance at a fixed frequency and the respective SP concentration.

## 3. Results and Discussions

### 3.1. Interaction Analysis Using Molecular Simulation

**Figure 2:**
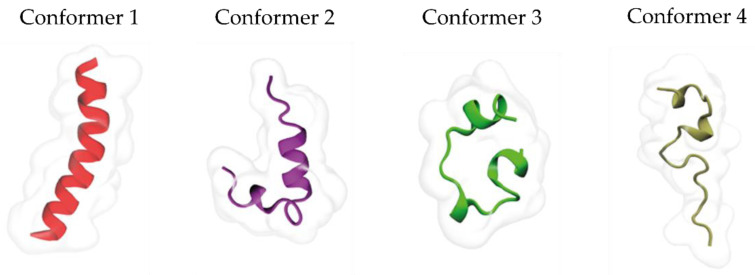
Selected conformers for the coupling simulation study.

Molecular dynamics were carried out to understand the interaction between ACE2*p* and ACE2 and assess their affinity and specificity. First, when the interaction between the peptide, as designed, and the protein is evaluated, the analyzed percentage of sequence identification between the ACE2*p* and ACE2 was low (29.17%). Therefore, the peptide was subjected to 500 ns of unrestricted molecular dynamics (MD) simulations to sample its different conformations better. During the first 100 ns, the ACE2*p* maintained its helicity structure. Then, it transitioned to random coil conformations. Conformational changes were systematically analyzed using RMSD-based clustering of the ACE2*p* backbone, employing a threshold of 2.5 Å, which allowed the identification of 10 clusters containing 69% of the sampled conformations (Table 1). One conformer was selected from the top four most populated clusters (Figure 2).

**Figure 3:**
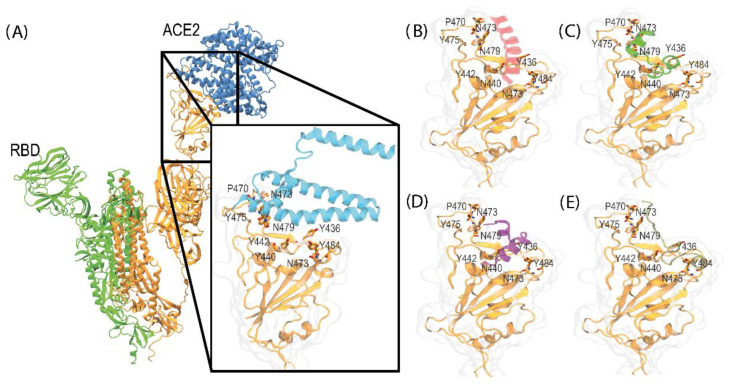
Representation of the binding site and the complexes formed between ACE2-RBD, obtained from the crystallographic structure (PDB-ID: 6CS2), and the different conformers of the ACE2 peptide-mimic complex generated through docking simulation using HADDOCK. (**A**) ACE2-RBD complex, (**B**) RBD–Conformer 1, (**C**) RBD–Conformer 2, (**D**) RBD–Conformer 3, and (**E**) RBD–Conformer 4.

The docking analysis of the ACE2*p* complex consistently resulted in a clamping conformation observed in the ACE2-RBD complex (PDB ID: 6CS2) in each docking simulation. These results are coherent and reflect ACE2 peptide-mimic’s high selectivity for this interaction binding site. The most favorable docking outcomes from the simulations with all the active residues of the RBD protein are detailed in Figure 3.

The docking simulations provide a valuable framework for understanding the interaction capability of the ACE2*p* peptide-mimetic with the RBD protein. This interaction could prevent the formation of the ACE2-RBD complex [37]. The results indicate a high peptide affinity for the receptor’s cavity, involving key residues such as Y436, P470, N473, Y475, N79, Y484, G488, G490, and Y491. These residues were previously identified as participating in hydrogen bond and hydrophobic interactions [43,44]. Moreover, these critical residues were consistently observed in the most favorable conformations, highlighting the relevance of this region for the interaction and the specificity of the peptide. The consistency of these residues across different simulations suggests that this contact region is essential for the effective recognition and binding of the peptide to the receptor, which could be linked to a key biological function.

### 3.2. Electrode Modification

**Figure 4:**
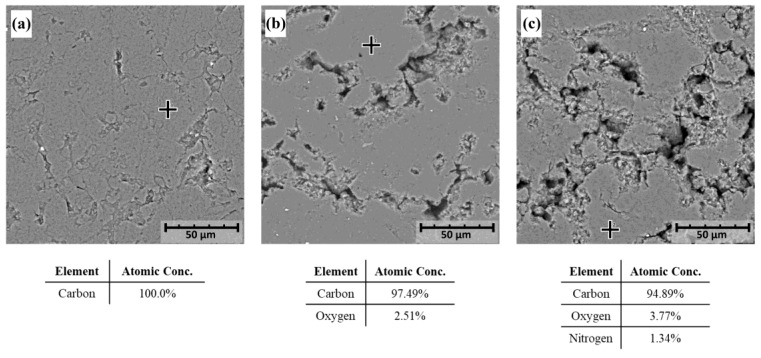
SEM/EDS analysis of three graphite electrodes: (**a**) blank graphite electrode (graphite), (**b**) electrografted graphite electrode (EG–graphite), and (**c**) peptide-modified graphite electrode (ACE2*p*–graphite). The crosses in each SEM image indicate the regions where the composition analysis was performed. The corresponding atomic concentrations of carbon, oxygen, and nitrogen are shown below each image. Scale bars represent 50 µm.

Scanning electron microscopy (SEM), coupled with energy-dispersive X-ray spectroscopy (EDS), was used to characterize the morphology and elemental composition of the graphite electrodes and its derivatives: a blank graphite electrode (graphite, Figure 4a), an electrografted graphite electrode (EG–graphite, Figure 4b), and a peptide-modified graphite electrode (ACE2*p*–graphite, Figure 4c).

Figure 4a shows a representative SEM image of the blank graphite electrode, which appears as a smooth, homogeneous surface interrupted by small fissures; these kinds of defects are characteristic of this type of multilayered materials [45,46] and are consistent with the expected morphology of pristine graphite electrodes. Further, the EDS analysis revealed that the electrode is composed of 100% carbon, consistent with its graphite composition. This confirms the high purity of the blank electrode and provides a baseline for comparison with the modified electrodes.

Figure 4b shows the SEM image of the electrografted graphite electrode, which displays a rough and irregular surface, which is attributed to the presence of the electrografted layer. The EDS analysis of the smooth zone (highlighted within the image) revealed that the electrografted layer contains 2.51% oxygen and 97.49% carbon of the underlying graphite electrode; the latter is consistent in multiple spots analyzed. Oxygen presence indicates successful grafting of the electroactive layer onto the graphite surface. The SEM image shows that the electrografted layer is well-adhered to the graphite surface, providing a stable platform for electrochemical sensing applications.

Figure 4c shows the SEM image of the peptide-modified graphite electrode, which exhibits a more complex surface topography due to the presence of the peptide layer. The EDS analysis performed on the highlighted zone indicated that the peptide-modified electrode contains 95.5% carbon, 3.2% oxygen, and 1.3% nitrogen, reflecting the incorporation of the peptide onto the electrode surface. The SEM image shows that the peptide layer is well-dispersed on the surface of the electrode, providing a high surface area for interaction with target analytes. The presence of nitrogen on the electrode surface suggests that the peptide layer is likely covalently bound to the electrografted surface.

**Figure 5:**
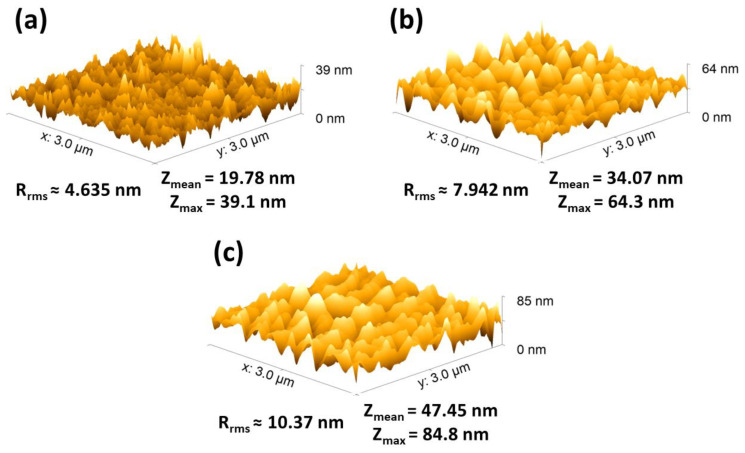
Comparison of electrode surfaces via AFM. (**a**) Blank graphite electrode (graphite), (**b**) electrografted graphite electrode (EG–graphite), and (**c**) ACE2*p*-modified graphite electrode (ACE2*p*–graphite).

The Atomic Force Microscopy (AFM) analysis, illustrated in Figure 5, provided insights into the morphology of the electrode surfaces, including measurements of average height, maximum height, and roughness factor (RMS). Specifically, the AFM images within this figure—Figure 5a–c—illustrate three different electrode conditions previously described. Figure 5a shows the blank electrode, with an average height of 19.78 nm and an RMS of 4.635. Further, Figure 5b corresponds to the electrografted graphite electrode, showing an average height of 34.07 nm, a maximum height of 64.3 nm, and an RMS of 7.942. Lastly, Figure 5c showcases the ACE2*p*-modified graphite electrode, with an average height of 47.45 nm, a maximum height of 84.8 nm, and an RMS of 10.37. The analysis of these data reveals a progressive increase in average height and roughness factor (RMS) as modifications are introduced to the electrode surface. This increase in height and roughness following modification with electrografting and subsequently with the peptide incorporation suggests successful adhesion and distribution of these layers on the electrode. The increased height and roughness observed in the ACE2*p*-modified electrode, detailed in Figure 5c, confirms the material’s incorporation and indicates a more complex and heterogeneous structure formation. These characteristics are beneficial for interactions with target analytes, as a larger surface area and more varied topography can improve the electrode’s sensitivity and selectivity [47]. Therefore, the AFM analysis presented in Figure 5 provides crucial evidence of surface modifications and offers insights into how these modifications might affect the electrode’s performance in detection applications.

**Figure 6:**
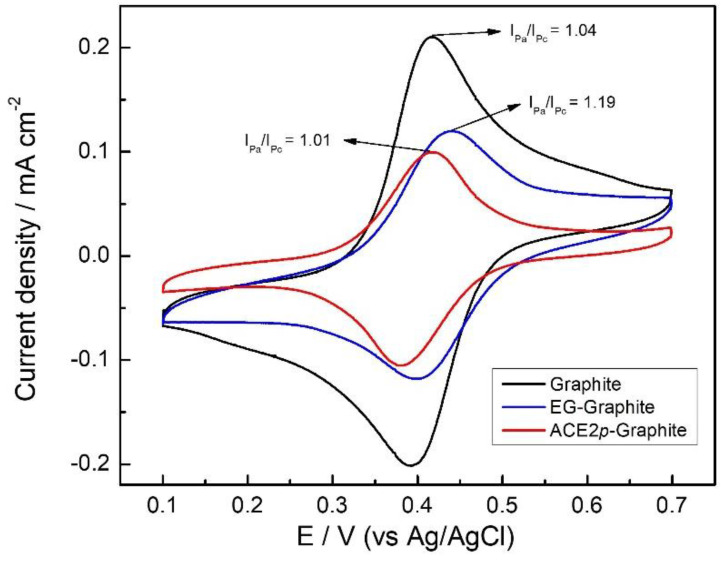
Cyclic voltammogram of [Fe(CN)_6_]^4−/3−^ 5 mM in PBS 1× pH 7.4 using graphite (black), EG-graphite (blue), and ACE2*p*-graphite (red) as the working electrode.

Figure 6 shows the cyclic voltammograms that evidence the electrochemical behavior of [Fe(CN)_6_]^−3/−4^ on graphite, EG–graphite, and ACE2*p*–graphite. [Fe(CN)_6_]^−3/−4^ redox couple is frequently used as a standard reversible redox couple [39,40]; therefore, I_Pa_/I_Pc_ for the system should be around 1 unless the surface of the electrode hinders diffusion or electron transfer processes. I_Pa_/I_Pc_ ratio for the three electrodes was found to be different. The blank graphite electrode had an I_Pa_/I_Pc_ ratio of 1.04, which suggests a reversible process, being coherent with a non-modified conductor surface. On the other hand, the EG–graphite electrode had an I_Pa_/I_Pc_ ratio of 1.19, indicating a less reversible process. Finally, including the peptide-mimic layer (ACE2*p*–graphite) increases the reversibility of the process, showing an I_Pa_/I_Pc_ ratio of 1.01. These results agree with the modification processes carried out on the graphite surface. For instance, the electrografting process supposes the inclusion of carboxylic acid residues, deprotonated at pH 7.4; these negatively charged species may hinder the diffusion of ferricyanide to the electrode due to electrostatic repulsions, leading to a less reversible process. On the other hand, the ACE2*p* used to modify the surface covalently links to the carboxylic residues, forming amide groups, neglecting the electrostatic repulsions previously discussed, and increasing the reversibility of the redox couple [48].

Regarding the peak currents, the ACE2*p*–graphite electrode showed a considerably lower peak current density than the other electrodes. The last is concordant with the inclusion of voluminous non-conductive species that obstacle diffusion of the redox active species to the electrode, resulting in smaller peak currents due to sluggish transport phenomena [49].

### 3.3. Detection of Recombinant SARS-CoV-2 Spike Protein RBD

Impedimetric detection of the spike protein (SP) on the ACE2*p*–graphite electrode was performed to evaluate the sensing capabilities of the modified surface through this technique. In this regard, numerous studies have demonstrated the advantages of employing total impedance for analyte detection. For example, several articles reported the successful application of electrochemical impedance spectroscopy (EIS) in detecting trace amounts of heavy metals, achieving low detection limits [50,51,52,53]. Furthermore, a growing body of literature supports the use of impedance-based techniques in conjunction with other analytical methods, such as surface plasmon resonance (SPR) [54] and quartz crystal microbalance (QCM) [55], to enhance the sensitivity and specificity of analyte detection. In electrochemical systems, the configuration involving three electrodes can be conceptualized as an analogous electrical circuit. This model incorporates elements such as the electrolytic solution’s ohmic resistance (R_s_), the Faradaic impedance (Z_f_), and the capacitance of the electrode–electrolyte interface, often referred to as double-layer capacitance (C_dl_), a construct detailed by Bard and Faulkner [56,57]. The absence of electrochemical activity at the electrode interface at open circuit potential renders the Faradaic processes dormant, leaving only the non-Faradaic processes contributing to the system’s impedance. Consequently, as depicted, the circuit’s complexity is reduced to a straightforward series arrangement of R_s_ and C_dl_. This reduction allows for a succinct expression of the system’s total impedance (Z) through the serial interconnection of R_s_ and C_dl_.

According to Yang et al. [58], the impedance behavior at lower frequencies (below 10 kHz) is dominated by the double-layer capacitance, significantly increasing the total impedance value and diminishing the relative importance of R_s_. This phenomenon delineates a capacitive domain within the double layer, enabling the detection of electrode impedance. Conversely, at frequencies exceeding 10 kHz, the double-layer capacitance’s impact on impedance becomes negligible, positioning R_s_ as the primary impedance contributor unaffected by frequency changes. This domain is characterized by ion conduction within the medium, marking it as the resistive phase.

**Figure 7:**
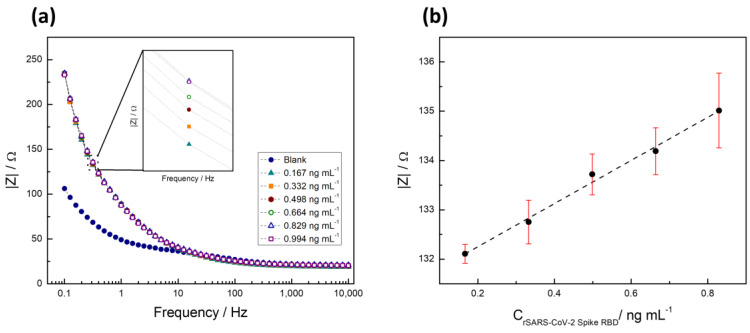
(**a**) Bode graph of a series of solutions with increasing concentration of recombinant 2019-nCoV spike RBD protein. (**b**) Variation of total impedance with respect to the concentration of recombinant 2019-nCoV spike RBD protein.

Thus, to quantify the recombinant 2019-nCoV spike RBD protein (SP), the total impedance of the system at a fixed frequency was obtained from the electrochemical impedance spectroscopy (EIS) data. Although Nyquist plots did not reveal significant differences (Figure S1), a clear change was observed in the frequency versus total impedance graph (Figure 7a). The frequency versus impedance analysis provided a more sensitive measure, revealing alterations in the electrode’s impedance behavior upon SP binding, which were not as apparent in the Nyquist plots. Figure 7a displays the Bode plot (frequency vs. impedance) of the recombinant protein established as a detection assay, highlighting an increase in the system impedance with rising protein concentration. This rise in impedance is attributed to the gradual blocking of the electrode surface by protein adsorption, which hinders electron transfer processes and leads to an increase in impedance values. The inset in Figure 7a provides a zoomed view of the points clustered at 0.316 Hz, a region identified with a better signal/concentration correlation.

In Figure 7b, the calibration curve, derived from measurements taken at a fixed frequency of 0.316 Hz across four assays, demonstrates a linear response and enhanced sensitivity within the concentration range of 0.167 ng mL^−1^ to 0.994 ng mL^−1^. This curve, constructed from total impedance measurements at a specified frequency, where the acquisition of each data point took approximately 3 min, emphasizes the system’s capability to detect the target protein within the specified concentration range efficiently.

To establish uniformity in the preparation and subsequent behavior of the electrodes, the calibration curve was performed in triplicate, consistently showing low error margins. This underscores the uniformity of ACE2*p* density on the electrode surface across various preparations and suggests that the peptide density does not critically influence the sensor’s functionality within the tested concentration range. Therefore, the observed electrochemical signals can be attributed to specific interactions between the immobilized peptide and the target spike protein. This ability to differentiate actual receptor binding from nonspecific adsorption is crucial for confirming the sensor’s effectiveness in accurately detecting the presence of SARS-CoV-2, aligning with the linear response and enhanced sensitivity observed in our calibration curve.

To further validate the specificity of our sensor, we evaluated the detection of the protein using an unmodified graphite electrode, as detailed in the Supplementary Information. Figure S1 demonstrates that the non-modified graphite electrode is not sensitive to the protein, indicating no significant correlation between increasing amounts of protein and changes in resistance. Consequently, any adsorption of the protein on the graphite surface would not affect the precise measurement of the protein on the modified electrode.

Building on the analytical insights garnered from the calibration curve and impedance measurements, the detection limit (LOD) and quantification limit (LOQ) were meticulously determined to be 45.08 pg mL^−1^ and 150.29 pg mL^−1^, respectively. These critical parameters were calculated employing the Miller and Miller method, which considers the standard deviation of the calibration curve to ensure precision in determining these limits [59]. This approach underscores the rigor in establishing the assay’s sensitivity and quantification capacity, highlighting its applicability in accurately detecting and quantifying the target protein within a defined concentration range.

The detection limit achieved underscores the method’s efficiency, supported by the efficient interaction between the ACE2*p* immobilized on the electrode surface and the target protein. At the assessed analyte concentrations, the impedance at 0.316 Hz maintains a linear correlation with concentration, as indicated by a high correlation coefficient (r^2^ > 0.989). This linear correlation facilitates precise analyte quantification at low concentrations, highlighting the method’s sensitivity in detecting the target protein through specific peptide–protein interactions.

The sensor’s observed sensitivity and rapid response can be further understood through molecular dynamics simulations (discussed earlier), which provide theoretical support for the experimental results. The simulations revealed stable binding interactions between the SARS-CoV-2 spike protein and the sensor’s surface, suggesting that the selected materials and experimental conditions promote high-affinity binding and effective signal transduction. This theoretical model helps explain the low detection limit achieved, as the simulated environment confirms efficient electron transfer at the interface, which aligns with the sensitivity observed in the experiments. Together, these findings underscore the sensor’s potential in spike protein detection and enhance our understanding of the factors contributing to its performance.

A recent study has successfully obtained the initial spike protein density data in SARS-CoV-2, but determining the number of identified viruses through the S protein calibration curve is currently not feasible [46]. Consequently, the data presented here were compared with existing electrochemical biosensors using spike protein as a target (Table 2). Recent developments in biosensor technology have yielded promising methods for detecting SARS-CoV-2 with high sensitivity and specificity. A comprehensive analysis of the current literature reveals a wide range of biosensors employing various electrochemical techniques for detection, such as cyclic voltammetry [33], chronoamperometry [33], differential pulse voltammetry [32], and the electrochemical impedance spectroscopy proposed here [30,34].

Notably, the proposed sensor achieved a detection limit of 45.08 pg mL^−1^, significantly lower than many previously reported values. For example, Mojsoska et al. (2021) developed an electrochemical immunosensor with a detection limit of 20 µg mL^−1^, requiring an incubation time of 45 min (Table 2), which, while effective for patient detection, presents limitations in sensitivity [60]. The approximately 400,000 times lower detection limit demonstrated here makes this sensor highly suitable for detecting trace amounts of SARS-CoV-2 spike protein in controlled settings. Additionally, this limit is lower than some previously reported values, such as the 18.2 ng mL^−1^ achieved using a modified electrode with gold nanoparticles reported by Soto and Orozco (2022) [30], and approaches other advanced sensors, like that reported by Sandoval et al. (2023), consisting of a modified interdigitated Au nanowire/antibody electrode, which reported a LOD of 0.14 pg mL^−1^ [34] (Table 2). The sensor proposed herein, however, offers high sensitivity alongside a rapid response time of only 3 min. This response time is substantially faster than previous sensors, whose times range from 15 to 45 min, and far surpasses the turnaround time for PCR. This rapid detection is critical for applications where timely results are essential.

**Table 2:** Electrochemical sensors based on detection of SARS-CoV-2 spike protein.

Detection Method	Working Electrode	Detection Range	Limit of Detection	Measurement Time	Reference
Differential pulse voltammetry	Chitosan-MoS_2_-reduced graphene oxide nanohybrid-modified glassy carbon electrode	10–100 ng mL⁻^1^	2.38 zg mL^−1^	15 min	[61]
Four-point Kelvin sensing system	Antibody-modified reduced graphene oxide (rGO) electrode	0.5–105 fg mL⁻^1^	0.5 fg mL^−1^	240 ms	[62]
Differential pulse voltammetry	Antibody-modified screen-printed graphene electrode (SPGE) with cellulose nanocrystals (CNCs)	0.1 pg mL⁻^1^–500 ng mL⁻^1^	2.0 fg mL^−1^	2 h	[63]
Differential pulse voltammetry	Antibody-modified graphene oxide with gold nanoparticles	10 fg mL⁻^1^–1 ng mL⁻^1^	1.0 fg mL^−1^	Not informed	[64]
Electrochemical impedance spectroscopy	Antibody-modified gold nanowire electrode	1 fg mL^–1^–1 μg mL^−1^	0.14 pg mL^−1^	20 min	[34]
Differential pulse voltammetry	Glassy carbon electrode modified with nitrogen-doped holey graphene	1 pg mL⁻^1^–10 ng mL⁻^1^	0.3 pg mL^−1^	60 min	[65]
Electrochemical impedance spectroscopy	Glassy carbon electrode modified with Fe_3_O_4_@SiO_2_-Au nanocomposites	0.1 ng mL^−1^–10 μg mL^−1^	4.78 pg mL^−1^	30 min	[66]
Square wave voltammetry	Carbon electrode modified with gold nanoparticles and an anti-SARS-CoV-2 antibody	250 pg mL^−1^–20 μg mL^−1^	36.3 pg mL^−1^	15 min	[67]
Electrochemical impedance spectroscopy	Graphite electrode modified with ACE2 peptide-mimic	0.167–0.994 ng mL^−1^	45.08 pg mL^−1^	3 min	This study
Square wave voltammetry	Graphene oxide-modified paper-based electrode.	1–1000 ng mL^−1^	0.11 ng mL^−1^	45 min	[68]
Electrochemical impedance spectroscopy	Screen-printed gold electrode functionalized with a thiolated peptide	0.05–1.0 μg mL^−1^	18.2 ng mL^−1^	15 min	[30]
Square wave voltammetry	Antibody-modified graphene electrode	Not informed	20 µg mL^−1^	45 min	[60]

Many electrochemical biosensors incorporate sophisticated methods for electrode preparation and modification, such as incorporating graphene oxide, gold nanoparticles/nanowires, or conductive polymers [30,32,33,34]. Although these methods offer the potential for enhanced performance, they can be challenging to implement and may be less suitable for large-scale production. Moreover, due to its straightforward manufacturing process (as depicted in Figure 1), reliance on standard materials, and the linear range obtained, the proposed sensor represents a promising platform for further improvement and optimization for detecting SARS-CoV-2 in complex biofluids or on surfaces.

In this line, these preliminary findings indicate no need for sample pretreatment to detect the spike protein, a step commonly observed in other electrochemical biosensors, as reported [30]. Regarding this point, in a previous study, the presence of certain osmolytes that could potentially influence the interactions between the peptide evaluated here and the protein was considered [36]. The potential interferences in peptide–protein binding arising from components commonly found in commercial kits used for saliva and nasal sample collection were thoroughly investigated in that study. These commercial kits typically employ a solution (buffer) to dilute biological samples (including bicarbonate, Tris, HEPES, and acetate) and osmolytes such as dimethyl sulfoxide (DMSO). Thus, the peptide behavior on those buffers was assessed, considering parameters such as pH and ionic strength (salt concentration). The results revealed that optimal conditions for these interactions were achieved at a basic pH, utilizing phosphate or bicarbonate buffers. Furthermore, the employment of DMSO, often used to enhance protein/DNA solubility and prevent peptide aggregation, showed that it could disrupt the interaction between ACE2*p* and the spike protein [36]. Therefore, the abovementioned factors could compromise the diagnostic systems that rely on this approach. In addition, the same work assessed the peptide interaction with nasopharyngeal swabs, classified as negative and positive for COVID-19, based on RT-qPCR analysis. The results demonstrated that highly diluted samples or those with low viral loads exhibited inadequate interaction with the peptide [36]. These observations suggest that the presence of components from commercial kits or the viral concentration might contribute to the detection limit of this technique. However, further testing is needed to fully assess the sensor’s performance in more complex matrices, such as fomites or biological samples. Nonetheless, the presented findings suggest that the proposed sensor has significant potential as a cost-effective and scalable tool for rapidly detecting SARS-CoV-2. Future studies will focus on evaluating its performance in various scenarios to facilitate effective and efficient detection of SARS-CoV-2.

## 4. Conclusions

In conclusion, the ACE2*p*-modified electrode developed in this study demonstrates a suitable linear range for detecting recombinant 2019-nCoV spike RBD protein (SP), with an impressive detection limit of 45.08 pg mL^−1^. The rapid sampling strategy allows a response time of only 3 min, making it highly promising for point-of-care diagnostic applications. Using total impedance as the analytical signal simplifies sample preparation and shortens sampling times. At the same time, the peptide-based sensor design allows for selective, sensitive detection across a broad range of analytes. This electrode was fabricated with accessible materials, and future modifications, such as adding nanostructures or miniaturizing components, could potentially enhance detection limits even further.

Molecular dynamics simulations provided additional insights into the interaction between the spike protein and the electrode surface under study conditions, indicating stable binding interactions. These findings help explain the high sensitivity observed experimentally, suggesting that the material and design choices in this sensor contribute significantly to its affinity and electron transfer efficiency.

Nevertheless, it is essential to acknowledge that the sensor has only been tested in a simulated environment using the SP as the analyte rather than the complete virus. While these initial results are encouraging, further testing in complex biological samples will be necessary to fully assess the sensor’s robustness and practicality for SARS-CoV-2 detection in clinical or environmental conditions. Future research will focus on validating and optimizing the sensor in diverse sample matrices, such as saliva or nasal swabs, to ensure reliable performance and mitigate potential interferences commonly found in these environments.

Overall, this method presents a cost-effective and efficient biosensor platform with significant potential for rapid SARS-CoV-2 detection, along with broader applications in point-of-care diagnostics.

## Figures and Tables

**Table 1:** Peptide-mimetic conformer distribution identified during the 500 ns MDs.

Cluster	Number of Conformers (Cutoff 2.5 Å)
1	170
2	168
3	94
4	85
5	34
6	34
7	32
8	27
9	24
10	22
Total	690

## Data Availability

Data are contained within the article and Supplementary Materials.

## Supplementary Materials

The following supporting information can be downloaded at: https://www.mdpi.com/article/10.3390/bios14120592/s1, Figure S1: Nyquist plot associated with detecting the recombinant Spike RBD protein at different concentrations on an unmodified graphite electrode.
