# Supplementary material for: Impedimetric Sensor for SARS-CoV-2 Spike Protein Detection: Performance Assessment with an ACE2 Peptide-Mimic/Graphite Interface
Source: Biosensors (Basel). 2024 Dec 3;14(12):592. doi: 10.3390/bios14120592 (PMC11674363; doi:10.3390/bios14120592)
Supplement: Supplementary file 1 [file biosensors-14-00592-s001.zip › biosensors-3339749-supplementary.pdf]

Supporting information

Detection: Performance Assessment with an ACE2 Peptide-Mimic/Graphite Interface

Diego Quezada [1], Beatriz Herrera [1], Rodrigo Santibáñez [1], Juan Luis Palma [2,3], Esteban Landaeta [2], Claudio A. Álvarez [4,5], Santiago Valenzuela [1], Kevin Cobos-Montes [6], David Ramírez [7], Paula A. Santana [1,*] and Manuel Ahumada [8,9,*]

[1]Instituto de Ciencias Aplicadas, Facultad de Ingeniería, Universidad Autónoma de Chile, el Llano
Subercaseaux 2801, San Miguel, Santiago 8910060, Chile; diego.quezada@uautonoma.cl (D.Q.);
beatriz.herrera@cloud.uautonoma.cl (B.H.); s.valenzuelamejias@icloud.com (S.V.); rodrigo.santibanez@cloud.uautonoma.cl (R.S.)
[2]School of Engineering, Universidad Central de Chile, Santiago 8330601, Chile; juan.palma@ucentral.cl (J.L.P.); esteban.landaeta@ucentral.cl (E.L.)
[3]Center for the Development of Nanoscience and Nanotechnology (CEDENNA), Santiago 9170124, Chile
[4]Laboratorio de Cultivo de Peces, Departamento de Acuicultura, Universidad Católica del Norte,
Coquimbo 1781421, Chile; claudio.alvarez@ucn.cl
[5]Laboratorio de Fisiología y Genética Marina, Centro de Estudios Avanzados en Zonas Áridas, Larrondo 1281 Coquimbo 1781421, Chile
[6]Departamento de Ciencias Químicas, Facultad de Ciencias Exactas, Universidad Andrés Bello,
Sede Concepción, Talcahuano 4260000, Chile; k.cobosmontes@uandresbello.edu
[7]Departamento de Farmacología, Facultad de Ciencias Biológicas, Universidad de Concepción,
Concepción 4030000, Chile; dramirezs@udec.cl
[8]Escuela de Biotecnología, Facultad de Ciencias, Ingeniería y Tecnología, Universidad Mayor, Camino La Pirámide 5750, Huechuraba, Santiago 8580745, Chile
[9]Centro de Nanotecnología Aplicada, Facultad de Ciencias, Ingeniería y Tecnología, Universidad Mayor, Camino La Pirámide 5750, Huechuraba, Santiago 8580745, Chile

* **Correspondence**: paula.santana@uautonoma.cl (P.A.S.); manuel.ahumada@umayor.cl (M.A.)

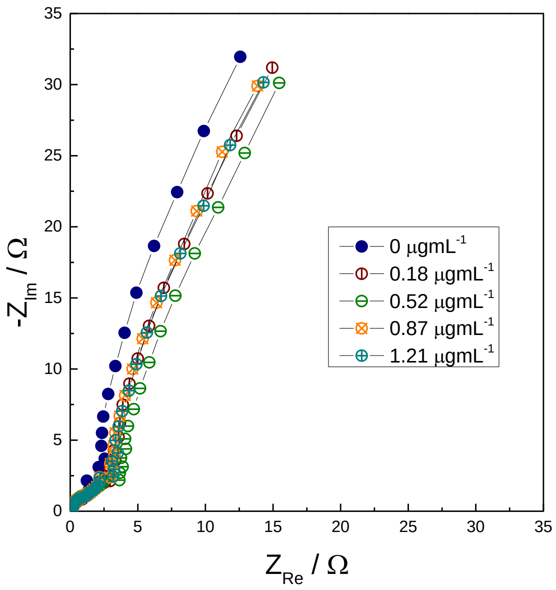

Figure S1. Nyquist plot associated with detecting the recombinant Spike RBD protein at different concentrations on an unmodified graphite electrode.
